# Genome Sequence of an *Acinetobacter baumannii* Strain Carrying Three Acquired Carbapenemase Genes

**DOI:** 10.1128/genomeA.01290-16

**Published:** 2016-11-17

**Authors:** Ken-Ichi Oinuma, Masato Suzuki, Kanako Sato, Kiyotaka Nakaie, Makoto Niki, Etsuko Takizawa, Mamiko Niki, Keigo Shibayama, Koichi Yamada, Hiroshi Kakeya, Yukihiro Kaneko

**Affiliations:** a Department of Bacteriology, Osaka City University Graduate School of Medicine, Osaka, Japan; b Department of Bacteriology II, National Institute of Infectious Diseases, Tokyo, Japan; c Department of Respiratory Medicine, Osaka City University Graduate School of Medicine, Osaka, Japan; d Department of Laboratory Medicine, Osaka City University Hospital, Osaka, Japan; e Department of Infection Control Science, Osaka City University Graduate School of Medicine, Osaka, Japan; f Research Center for Infectious Disease Sciences, Osaka City University Graduate School of Medicine, Osaka, Japan

## Abstract

The emergence of multiple-carbapenemase-producing *Acinetobacter* strains has been a serious concern during the past decade. Here, we report the draft genome sequence of an *Acinetobacter baumannii* strain isolated from a Japanese patient with three acquired carbapenemase genes: *bla*_NDM-1_, *bla*_TMB-1_, and *bla*_OXA-58_.

## GENOME ANNOUNCEMENT

Some members of the genus *Acinetobacter* effectively acquire antimicrobial resistance through a variety of different mechanisms. One of the most problematic methods of antimicrobial resistance is the acquisition of carbapenemase genes. Carbapenemase gene products can hydrolyze a broad range of β-lactams, including carbapenems. There are several different types of carbapenemases, such as metallo-β-lactamase (including NDM types, IMP types, and VIM types) and some oxacillinase-type enzymes (including OXA-23, OXA-24, and OXA-58) in *Acinetobacter* species isolates (1), and the list of carbapenemases continues to expand. One example of a newly found carbapenemase is Tripoli metallo-β-lactamase 1 (TMB-1) (2, 3). The TMB-1 gene was first identified in an *Achromobacter xylosoxidans* strain isolated in Tripoli, Libya, in 2012. In addition to TMB-1, the derivative, TMB-2, was also found in non-*baumannii Acinetobacter* spp. isolated in Japan (4, 5). Carbapenem-resistant *Acinetobacter* strains possessing a single acquired carbapenemase gene have become widespread throughout the world. During the past decade, multiple carbapenemase producers have emerged (6). Multiple carbapenemase-containing strains raise a serious concern because they tend to show higher β-lactam resistance, leaving fewer treatment options.

Here, we announce the draft genome sequence of an *A. baumannii* strain, OCU_Ac16a. OCU_Ac16a was recently isolated by intratracheal aspiration of a 74-year-old male patient with esophageal cancer at Osaka City University Hospital. Based on our records, the patient did not travel internationally in the several months before admission. The strain was found to be resistant to carbapenems, according to the 2016 CLSI breakpoints (7). Whole-genome sequencing with a 400- to 800-bp insert size was performed using the MiSeq system (Illumina). Paired-end reads (2 × 300 bp) were assembled *de novo* using the CLC Genomics Workbench version 8.5.1 (Qiagen).

The draft genome sequence of this isolate consists of 192 contigs, a total size of 4,071,967 bp, and an *N*_50_ value of 87,985 bp. The mean G+C content was 38.7%. A total of 4,025 coding genes were annotated using the RAST toolkit on the PATRIC server (https://www.patricbrc.org/). Antimicrobial resistance genes were detected using ResFinder version 2.1 (http://cge.cbs.dtu.dk/services/ResFinder/). Surprisingly, OCU_Ac16a carried five different β-lactam resistance genes, including two metallo-β-lactamases (NDM-1 and TMB-1), two oxacillinases (OXA-58 and OXA-208), and an AmpC β-lactamase (ADC-25 variant) gene. Of note, OXA-208, a variant of OXA-51, and three of the acquired β-lactamases, NDM-1, TMB-1, and OXA-58, are enzymes with carbapenem-hydrolyzing activity (1–5).

The emergence of *Acinetobacter* strains carrying two acquired carbapenemase genes has been limited to a limited number of countries, such as Greece (6). To the best of our knowledge, this is the first report of an *Acinetobacter* strain carrying three acquired carbapenemase genes. Detailed data, including the transmissibility of the carbapenemase genes, will be reported in a future publication.

### Accession number(s).

This whole-genome shotgun sequencing project has been deposited in DDBJ/EMBL/GenBank under the accession numbers BDHK01000001 to BDHK01000192. The version described in this report is the first version.
